# Supplementary material for: Conserved Metastatic Cell States and Spatial Immune Microenvironment Remodeling Define Pancreatic Cancer Liver Metastases
Source: bioRxiv. 2026 Sep 20:2026.05.27.728281. Preprint. [Version 2] doi: 10.64898/2026.05.27.728281 (PMC13596145; doi:10.64898/2026.05.27.728281)
Supplement: Supplementary file 1 [file NIHPP2026.05.27.728281V2-supplement-1.pdf]

bioRxiv preprint doi: https://doi.org/10.64898/2026.05.27.728281; this version posted September 20, 2026. The copyright holder for this preprint (which was not certified by peer review) is the author/funder, who has granted bioRxiv a license to display the preprint in perpetuity. It is made available under a CC-BY 4.0 International license.

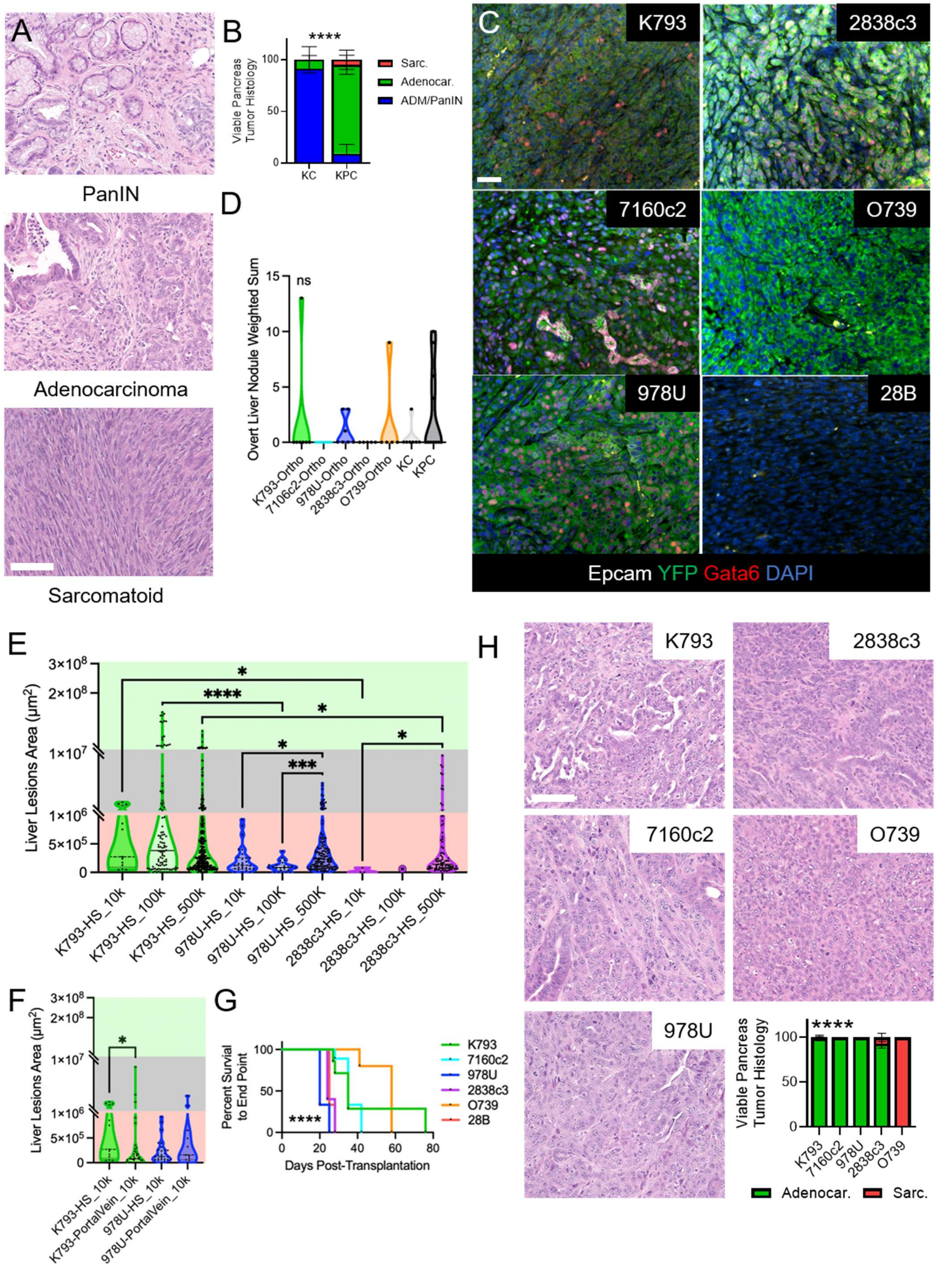

bioRxiv preprint doi: https://doi.org/10.64898/2026.05.27.728281; this version posted September 20, 2026. The copyright holder for this preprint (which was not certified by peer review) is the author/funder, who has granted bioRxiv a license to display the preprint in perpetuity. It is made available under a CC-BY 4.0 International license.

Supplementary Figure 1: Cancer cell-intrinsic differences define liver metastatic capacity

A. Representative histopathology micrographs from spontaneous mouse models. Scale 100 µm. **B**. Relative percentage of histological subtypes in the viable pancreatic cancer regions from the spontaneous KC and KPC mouse models. n = KC: 7, KPC: 10 mice. Two-way ANOVA. Multiple comparisons showed significant differences in PanIN and Adenocarcinoma between the genotypes. Variation of penetrance between the sarcomatoid resulted in non-significance. **C.** Representative micrographs of Epcam, YFP, and Gata6 immunolabeling of orthotopic tumors. Scale 20 µm. Note 28B is a YFP^- cell line. **D**. Weighted sum by binned size of overt liver nodules of orthotopically injected (ortho). n = K793:7, 7160c2: 9, 978U: 7, 2838c3: 5, O739: 5, KC: 7, KPC: 10 mice. **E-F**. Quantification of each metastatic liver nodule area of hemisplenic (F) or direct portal vein (G) transplanted cell lines. Axis breaks correspond to clinical imaging groups of micrometastases (red), borderline detectable (gray), and detectable (green). **G**. Percent survival to endpoint of orthotopic transplants. n =K793:7, 7160c2: 9, 978U: 6, 2838c3: 5, O739: 5, 28B: 3 mice. Mantel-Cox test. **H**. Representative micrographs and quantification of histopathology of pancreatic tumors in dual transplants and relative percentage of histological type in the viable pancreatic cancer regions of dual transplanted tumors. n = K793:9, 7160c2: 6, 978U: 6, 2838c3: 5, O739: 6. Two-way ANOVA. Multiple comparisons showed each of the predominantly adenocarcinoma lines was significantly different from O739. Significance determined by Kruskal-Wallis One-Way ANOVA, unless indicated otherwise. * $p<0.05$, ** $p<0.01$, *** $p<0.001$, **** $p<0.0001$, ns, not significant.

bioRxiv preprint doi: https://doi.org/10.64898/2026.05.27.728281; this version posted September 20, 2026. The copyright holder for this preprint (which was not certified by peer review) is the author/funder, who has granted bioRxiv a license to display the preprint in perpetuity. It is made available under a CC-BY 4.0 International license.

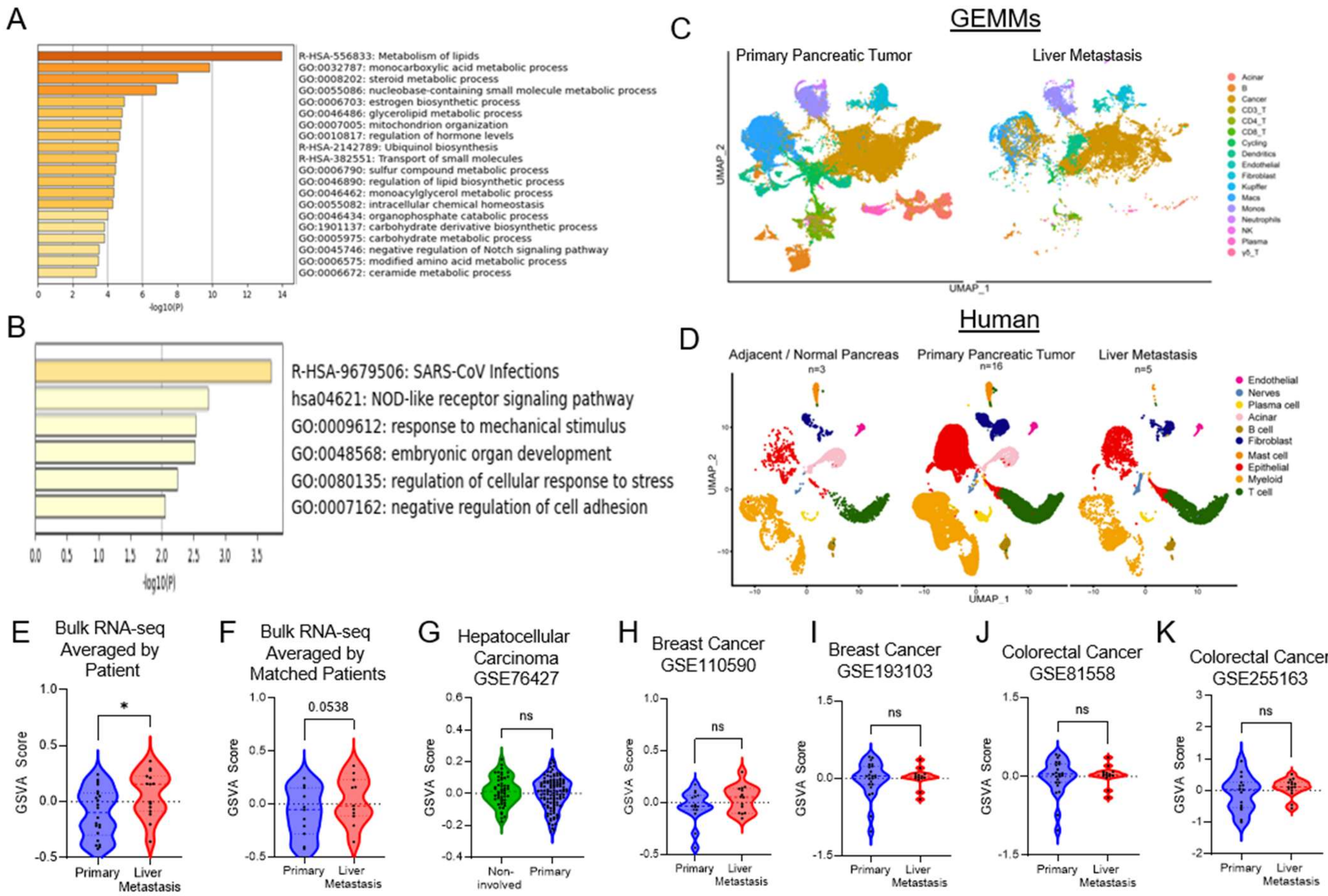

Supplementary Figure 2: A Conserved Metastatic Cell State Is Enriched in PDAC Liver Metastases.

A-B. Metascape functional enrichment analysis of the commonly upregulated genes in the high-liver-tropic (A) and low liver-tropic (B) gene signatures. **C**. UMAP visualization of scRNA-seq data showing major cell populations across GEMM. **D.** UMAP visualization of scRNA-seq data showing major cell populations across human PDAC samples. **E-F**. GSVA score of the High-Tropic signature in bulk RNA-seq of all samples from rapid autopsy, averaged by patient (E) and showing only matched primary and liver metastases (F). Unpaired T test or Paired T test, respectively. **G-K**. GSVA score of the High-Tropic signature in bulk RNA-seq of Hepatocellular Carcinoma dataset GSE76427 of matched non-involved liver and the primary (liver) tumor (G),

bioRxiv preprint doi: https://doi.org/10.64898/2026.05.27.728281; this version posted September 20, 2026. The copyright holder for this preprint (which was not certified by peer review) is the author/funder, who has granted bioRxiv a license to display the preprint in perpetuity. It is made available under a CC-BY 4.0 International license.

Breast cancer datasets GSE110590 (H) and GSE193103 (I), Colorectal cancer datasets GSE81558 (J) and GSE255163 (K). Unpaired T test with Welch's correction (G, H and I). * $p<0.05$, *** $p<0.001$, **** $p<0.0001$, ns, not significant.

bioRxiv preprint doi: https://doi.org/10.64898/2026.05.27.728281; this version posted September 20, 2026. The copyright holder for this preprint (which was not certified by peer review) is the author/funder, who has granted bioRxiv a license to display the preprint in perpetuity. It is made available under a CC-BY 4.0 International license.

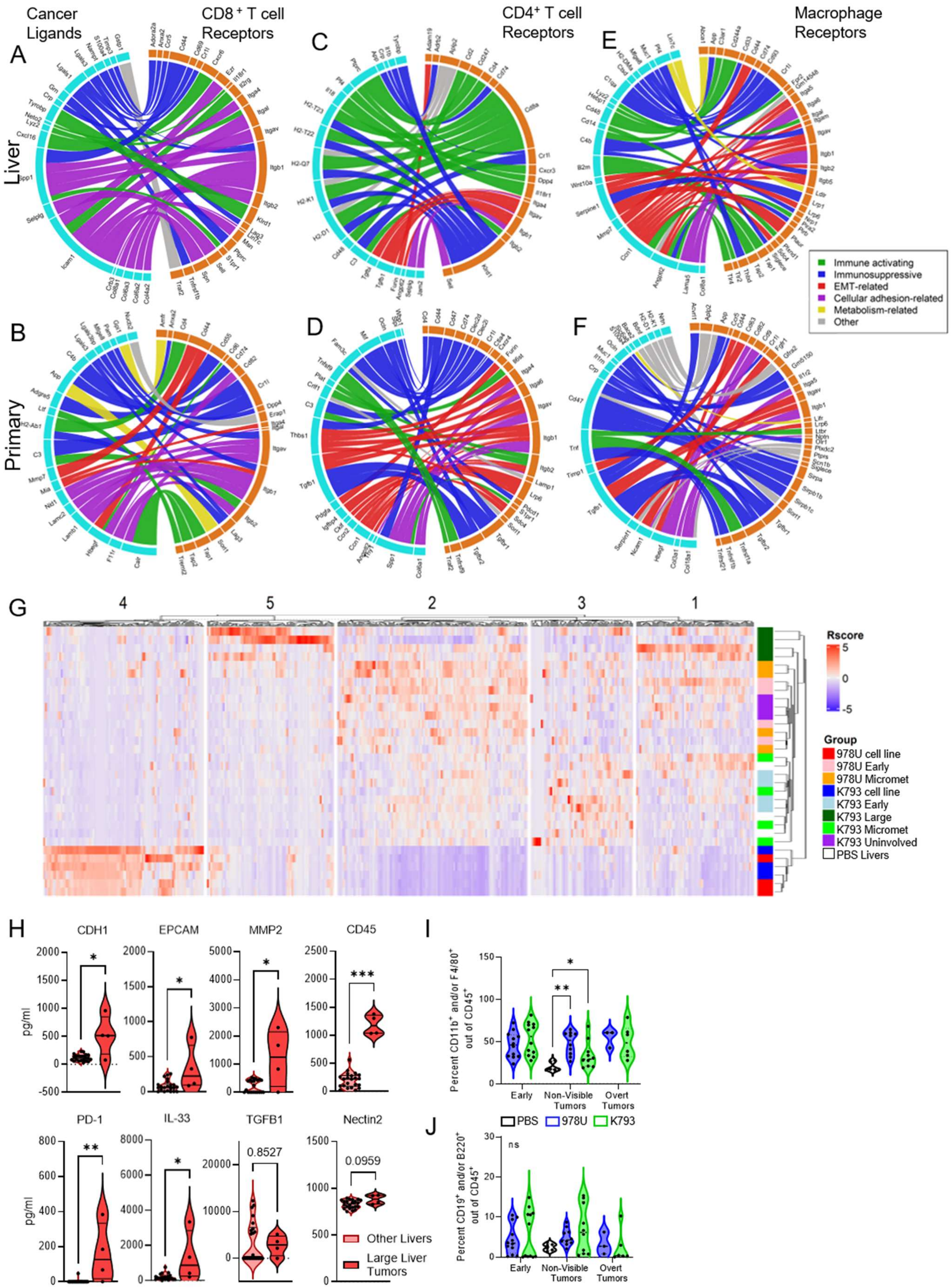

bioRxiv preprint doi: https://doi.org/10.64898/2026.05.27.728281; this version posted September 20, 2026. The copyright holder for this preprint (which was not certified by peer review) is the author/funder, who has granted bioRxiv a license to display the preprint in perpetuity. It is made available under a CC-BY 4.0 International license.

Supplementary Figure 3: Cross-Species Analyses Reveal Immunomodulatory Signaling in PDAC Liver Metastases.

A-F. Predicted ligand–receptor interactions between cancer cells and immune populations across PDAC GEMM samples. Interactions between cancer cells and $CD8^+$ T cells in liver metastases (A) and primary pancreatic tumors (B), cancer cells and $CD4^+$ T cells in liver metastases (C) and primary pancreatic tumors (D), and cancer cells and macrophages in liver metastases (E) and primary pancreatic tumors (F). **G**. Unbiased hierarchical clustering heatmap of cell lines and tumor samples based on a murine cytokine array, with k-means clustering. **H.** Violin plots showing the mean expression of ligand-receptor associated proteins identified in liver metastatic cancer cells through NicheNet analyses across liver samples. Mann-Whitney t test. **I-J**. Flow cytometric quantification of $CD11b^+$ and/or $F4/80^+$ macrophages (I), and B cells (J) out of viable $CD45^+$ cells across hemisplenic transplanted liver. Two-way ANOVA. * $p<0.05$, ** $p<0.01$, *** $p<0.001$, ns, not significant.

bioRxiv preprint doi: https://doi.org/10.64898/2026.05.27.728281; this version posted September 20, 2026. The copyright holder for this preprint (which was not certified by peer review) is the author/funder, who has granted bioRxiv a license to display the preprint in perpetuity. It is made available under a CC-BY 4.0 International license.

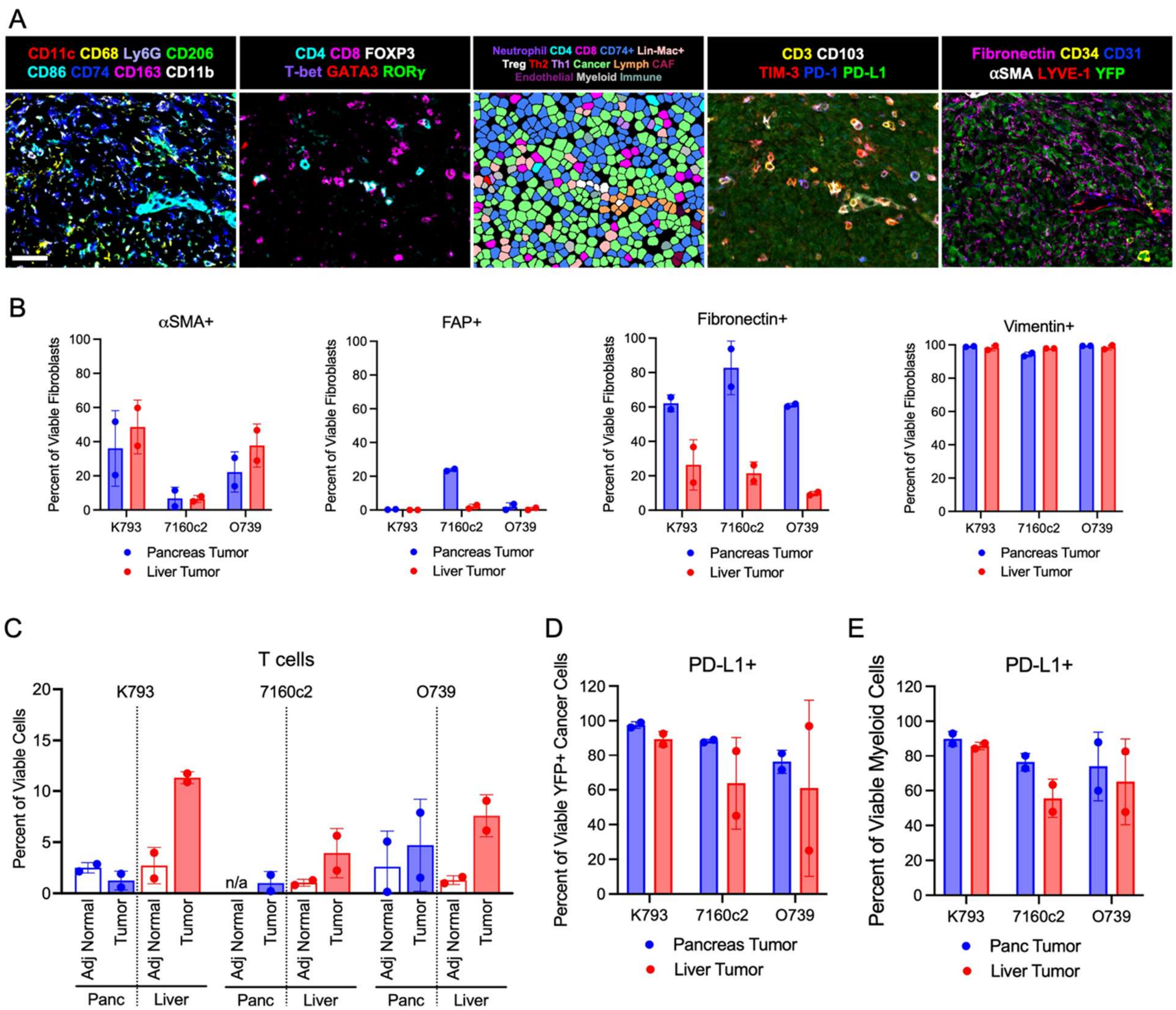

bioRxiv preprint doi: https://doi.org/10.64898/2026.05.27.728281; this version posted September 20, 2026. The copyright holder for this preprint (which was not certified by peer review) is the author/funder, who has granted bioRxiv a license to display the preprint in perpetuity. It is made available under a CC-BY 4.0 International license.

Supplementary Figure 4: Anatomical Site Is a Major Determinant of PDAC Immune Composition.

A. Representative micrographs of the discussed immune phenotypic markers and cancer and stroma (scale 20 μm). The middle panel displays cell phenotype overlays representing the discussed populations. **B**. Frequency of each CAF marker (αSMA, FAP, Fibronectin, Vimentin) out of viable fibroblasts according to the tumor ROI of each cell line. **C**. Frequency of T cells out of all viable cells according to ROI. **D**. PD-L1 expression of viable YFP^{+} cancer cells according to tumor ROIs. **E**. PD-L1 expression of viable myeloid cells (CD45^{+}, CD19^{-}, CD3^{-}, NCR1^{-}) according to tumor ROIs. n = 2 mice for all groups.

bioRxiv preprint doi: https://doi.org/10.64898/2026.05.27.728281; this version posted September 20, 2026. The copyright holder for this preprint (which was not certified by peer review) is the author/funder, who has granted bioRxiv a license to display the preprint in perpetuity. It is made available under a CC-BY 4.0 International license.

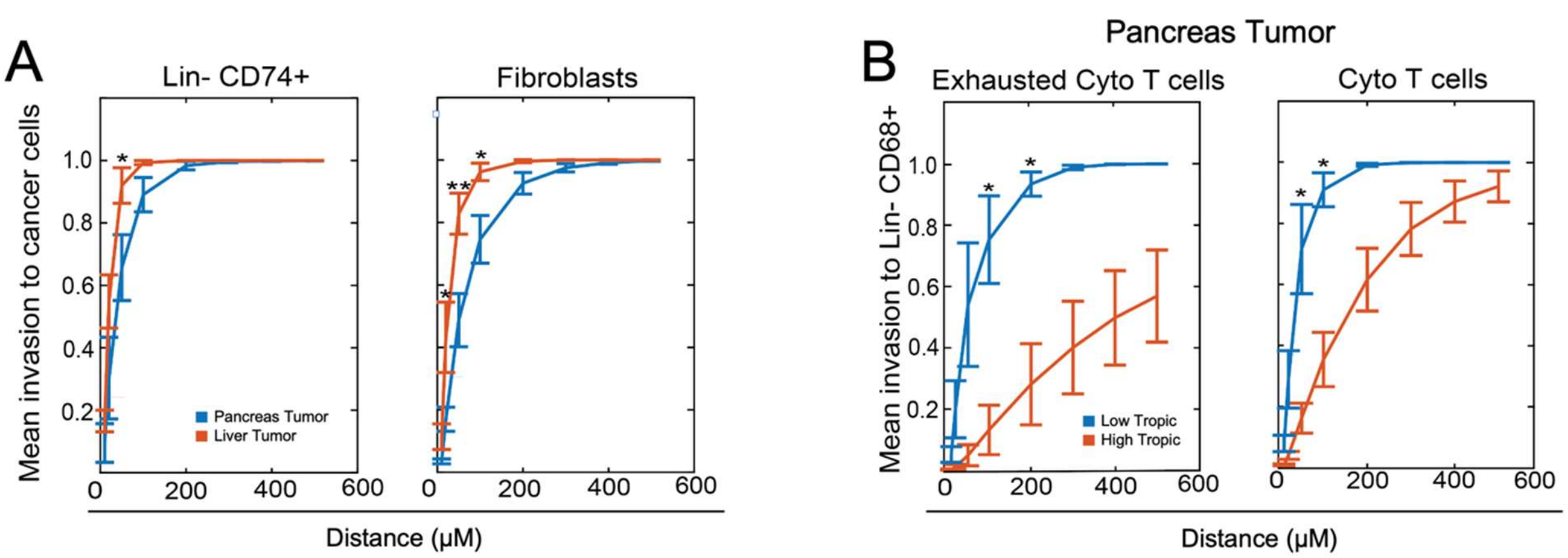

Supplementary Figure 5: Liver tumors display enhanced TME infiltration around cancer cells.

A. G function plots showing mean infiltration to cancer cells of Lin^{-} $CD74^{+}$ (APC) cells and fibroblasts within pancreas and liver tumors. **B**. G function plots showing mean infiltration to Lin^{-} $CD68^{+}$ cells from exhausted ($PD\text{-}1^{+}$ $TIM3^{+}$) cytotoxic T cells and total cytotoxic T cells within

bioRxiv preprint doi: https://doi.org/10.64898/2026.05.27.728281; this version posted September 20, 2026. The copyright holder for this preprint (which was not certified by peer review) is the author/funder, who has granted bioRxiv a license to display the preprint in perpetuity. It is made available under a CC-BY 4.0 International license.

pancreas tumors of high liver-tropic cell lines (K793 and 7160c2) and low-liver tropic cell line (O739). Two-sample, two-sided t-tests. * $p<0.05$, ** $p<0.01$.

bioRxiv preprint doi: https://doi.org/10.64898/2026.05.27.728281; this version posted September 20, 2026. The copyright holder for this preprint (which was not certified by peer review) is the author/funder, who has granted bioRxiv a license to display the preprint in perpetuity. It is made available under a CC-BY 4.0 International license.

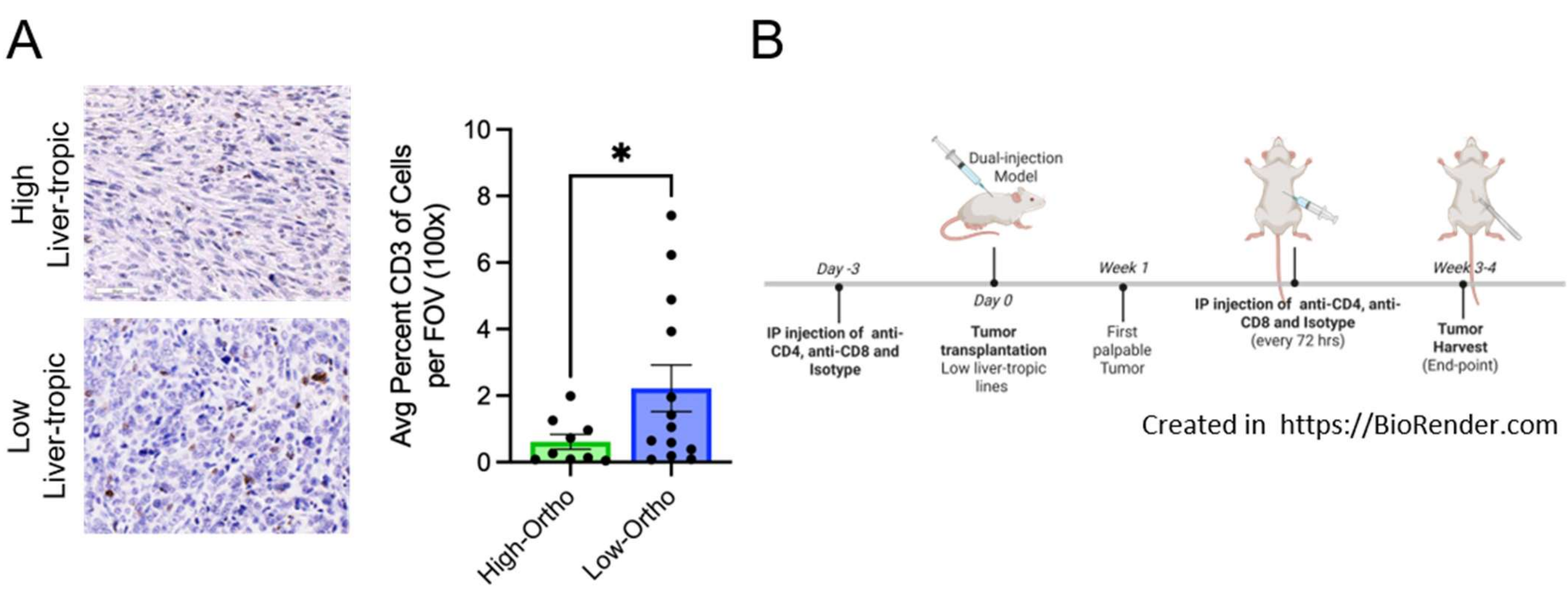

bioRxiv preprint doi: https://doi.org/10.64898/2026.05.27.728281; this version posted September 20, 2026. The copyright holder for this preprint (which was not certified by peer review) is the author/funder, who has granted bioRxiv a license to display the preprint in perpetuity. It is made available under a CC-BY 4.0 International license.

Supplementary Figure 6: T cells constrain PDAC liver metastatic outgrowth.

A. Representative CD3 IHC micrographs (scale 50μm) and quantification of average percent $CD3^+$ cells per 100x FOV. n= High lines: 9, Low lines: 13 mice. Average of 7 FOV per mouse. Data are ± Standard Error Mean (SEM). Unpaired t test with Welch's correction. **B**. Schematic of T cell depletion study.

bioRxiv preprint doi: https://doi.org/10.64898/2026.05.27.728281; this version posted September 20, 2026. The copyright holder for this preprint (which was not certified by peer review) is the author/funder, who has granted bioRxiv a license to display the preprint in perpetuity. It is made available under a CC-BY 4.0 International license.

Tables List

Supplementary Table 1. Cell line characteristics.

Supplementary Table 2. EMT gene signature

Supplementary Table 3. Overt Incidences at Necropsy by mouse ChiSquare Analysis Results

Supplementary Table 4. Microscopic Incidences by mouse ChiSquare Analysis Results

Supplementary Table 5. High Liver-Tropic Gene Signature

Supplementary Table 6. Low Liver-Tropic Gene Signature

bioRxiv preprint doi: https://doi.org/10.64898/2026.05.27.728281; this version posted September 20, 2026. The copyright holder for this preprint (which was not certified by peer review) is the author/funder, who has granted bioRxiv a license to display the preprint in perpetuity. It is made available under a CC-BY 4.0 International license.

Supplementary Table 7. COMET Antibody Staining Panel

Supplementary Table 8. Mouse Cohorts

Supplementary Table 9. Flow Cytometry Antibody Panel / Gating – T cell, B cell, Myeloid

Supplementary Table 10. Flow Cytometry Antibody Panel / Gating – Myeloid/Lymphoid

Supplementary Table 11. Flow Cytometry Antibody Panel / Gating – CD4 Subsets

Supplementary Table 12. Flow Cytometry Antibody Panel / Gating – CD8 Cell Status

Supplementary Table 13. Flow Cytometry Antibody Panel / Gating – T cell Depletion
